# Robust LiDAR-Based Train Detection via Point Cloud Segmentation for Railway Safety

**DOI:** 10.3390/s26051514

**Published:** 2026-02-27

**Authors:** Yuxing Yang, Siyue Yu, Jimin Xiao

**Affiliations:** 1 School of Information and Communications Engineering, Xi’an Jiaotong University, Xi’an 710000, China; 2 Department of Intelligent Science, School of Advanced Technology, Xi’an Jiaotong-Liverpool University, Suzhou 215000, China

**Keywords:** point cloud segmentation, robust train detection, railway safety monitoring

## Abstract

Ensuring railway safety requires reliable monitoring of trains in critical safety areas, such as station throat zones and railway crossings. Compared with cameras, roadside LiDAR can more reliably capture the geometry of trains under low-light, high-speed, and adverse weather conditions. However, industrial LiDAR solutions still primarily use the background comparison technique, which compares each sample against a pre-recorded clean map and then applies a size-based filter. Such approaches are highly sensitive to point cloud background changes arising from varying LiDAR installation distances, train speeds, and surface materials, often resulting in fragmented clustering and missed detections. In this paper, train detection is reformulated as a point-level semantic segmentation problem. A lightweight 3D segmentation network that directly predicts train points from raw data is designed, and clustering-based post-processing is applied to generate train-level events in real time. Experiments on real railway data under various operating conditions show that the proposed method achieves higher detection accuracy and greater robustness than traditional compare-based methods and representative deep learning benchmark methods, and is therefore suitable for practical railway safety monitoring.

## 1. Introduction

Reliable monitoring along the railway line, such as hillsides, tunnels, and flyovers, is important for rail safety [1,2,3,4]. Using effective sensors to monitor along railway lines can effectively ensure train safety and prevent rockfalls, landslides, and mudslides. Train detection refers to the task of automatically identifying, locating, and tracking moving trains. It can provide core information on whether the track line is occupied and issue collision warnings. Currently, camera-based surveillance systems are widely used in practice [5,6]. However, under low illumination and adverse weather conditions, detection performance would decrease significantly [7,8]. Meanwhile, captured images become blurry at high speeds, making it difficult to reliably detect and localize the train. In contrast, light detection and ranging (LiDAR), when deployed at the same positions as cameras, can capture train point clouds with full geometric information, and the data quality is largely unaffected by ambient light. Therefore, LiDAR-based surveillance systems are a promising solution for railway safety monitoring.

Existing industrial-grade LiDAR-based train detection systems typically use background comparison methods [9,10]. The first step is to create a sufficiently clean background: combining multiple consecutive point cloud frames captured under clear weather conditions to form a voxel-level template. Subsequently, test data undergoes pre-processing (voxel downsampling, region cropping, and outlier removal) before being individually compared against the clean background. Anomaly points not present in the background are filtered based on distance thresholds. These anomalies are then clustered into several abnormal point cloud clusters. Then, these clusters are filtered using predefined geometric rules, such as volume, dimension sizes (length, width, and height), or relative position to the track. The retained abnormal clusters are represented as the point cloud data of the trains.

**Figure 1:**
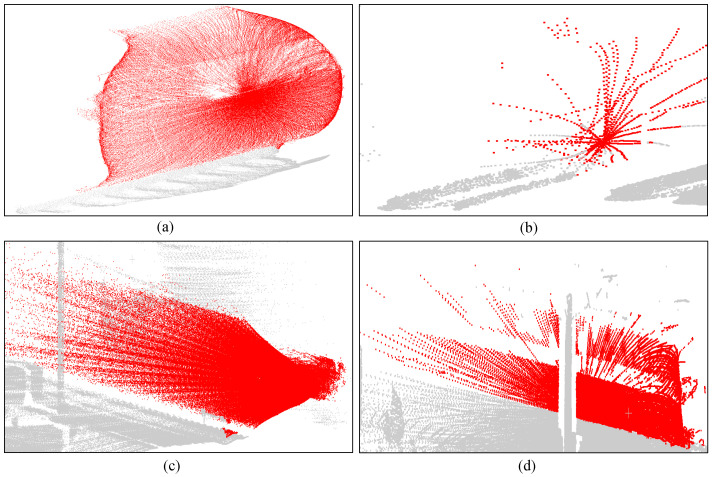
Point cloud data with different geometric structures. Red and grey points represent train and background points. (**a**) Dense form. (**b**) Sparse form. (**c**) High-speed running. (**d**) Obscured form.

However, background comparison methods have several limitations [3]. Firstly, the approach makes an implicit and critical assumption: the background environment remains static over time. This is essential for accurately distinguishing foreground trains from the background. However, LiDAR sensor positions may shift, and vegetation in the background may undergo temporal changes. Both these factors can change the background environment, thereby affecting detection results. Secondly, clean background data is collected under sunny weather conditions. Thus, detection performance in adverse weather conditions such as rain, fog, or snow may also be significantly affected. Most critically, the geometric structure of train point cloud data varies. As shown in Figure 1, different LiDAR sensor positions across scenarios may yield point clouds that are either excessively dense or sparse. Train speed also influences the point cloud structure. Furthermore, object occlusions and variations in the train’s material reflectivity similarly affect its structural representation. Consequently, the detection pipeline based on background comparison methods has the following issues: abnormal clusters may be segmented into multiple sub-abnormal clusters, which are filtered out due to insufficient size, leading to missing detections. Meanwhile, environmental noise can cause false positives. This not only affects the accuracy of train detection but also impairs the generalization capability of the method across different scenarios.

Unlike background comparison methods, we treat train detection as a LiDAR-based point cloud segmentation problem. The surveillance system processes captured points from the railway scene in real time. A three-dimensional point cloud segmentation neural network predicts point-level results and aggregates into object-level events [11,12]. Compared to background comparison methods, point cloud segmentation-based methods enable direct train detection assessment on acquired point cloud data. And it adapts to variations in train point cloud structure across different locations, installation distances, and speeds, thereby achieving more stable and generalizable train detection performance [13,14].

Extensive experiments have been conducted to evaluate the performance of the proposed framework in train detection. The industrial background comparison method is assessed against the proposed point cloud segmentation-based approach in terms of detection accuracy and computational cost. We also compared our segmentation results with those of other deep learning point cloud segmentation methods. Additionally, the impact of pre-processing, model, and post-processing parameters on segmentation and train detection results is analysed. Visualization of predicted train point cloud data is also provided for discussion. The main contributions of this work are as follows.

- The railway train detection task is reformulated from a comparison-based paradigm to a segmentation-driven framework to enhance the semantic understanding of railway scenarios.
- A geometry-aware post-processing method, which combines rail-region constraints and distance-based filtering, is introduced to improve the detection stability under background disturbance and adverse weather conditions.
- The proposed framework can identify different types of train samples under different weather and lighting conditions, with empirical evidence provided through cross-scene and adverse-condition evaluations.
- Extensive experimental evaluations on train detection efficiency, accuracy, and robustness demonstrate that the proposed framework can achieve a balance between detection performance and operational requirements.

The structure of this paper is as follows: Section 2 reviews several recent works on train detection for railway safety and point cloud segmentation for transportation scenarios. An overview of the proposed point cloud segmentation-based train detection framework is presented in Section 3. Section 4 discusses the evaluation metric and presents experimental results for point cloud segmentation and train detection accuracy, and efficiency. Finally, Section 5 concludes the paper and provides potential directions for future research.

## 2. Related Work

### 2.1. Train Detection for Railway Safety

Train detection is a core task in railway safety monitoring systems. It has attracted significant research attention. It can effectively provide position information on trains, thereby ensuring the track is in the correct occupied state and preventing collisions. Currently, traditional industrial train detection methods primarily rely on sensor technologies. A comprehensive review of sensing techniques and detection methods for train approach detection was conducted in [15], discussing the application of mechanical (measuring the deformation caused by the train’s axles), magnetic (detecting the changes in the electromagnetic field when metal objects pass by), and acoustic (measuring the sound waves on the track when the train passes) sensors in train detection. A novel method of installing a force sensor on the rail sleeper was proposed in [16]. By measuring the vibration signal, the speed and wheelbase of the train passing can be estimated in real time. This method can be used in an electromagnetic interference environment, but it still relies heavily on the specific structure of the rail sleeper and has limited generalizability. A real-time perception and warning train safety monitoring system based on distributed sensors and cloud analysis was proposed in [17]. These methods all utilize physical sensors to provide monitoring signals for train detection, deployment, installation, and maintenance, which are very costly [15,18].

**Figure 2:**
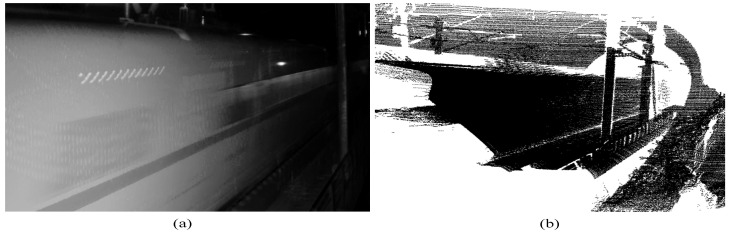
Representative multi-sensor train data: (**a**) camera-based image; (**b**) LiDAR-based point cloud data.

Nowadays, non-intrusive computer vision solutions and machine learning algorithms are also widely used in railway safety monitoring [19,20]. The captured train images and point cloud data are shown in Figure 2. In [21], a deep learning-based monitoring system for railway-level crossings is proposed. It processes the video data captured by the camera and trains it in a convolutional neural network to determine whether the level crossings are occupied. Although this method has high accuracy, it performs poorly in low-light conditions and during high-speed movement. Therefore, many studies have used LiDAR to collect 3D point cloud data for train detection and scene understanding. A railway-scene dataset containing only point cloud data was constructed in [22] to study weakly supervised semantic segmentation. Graph convolutional neural networks for point cloud segmentation in railway environments were utilized in [23]. Similarly, our method focuses solely on 3D point cloud data, treating train detection as a downstream task of point cloud segmentation, and uses deep learning models to accurately and efficiently complete the task.

### 2.2. Background Comparison for Transportation Scenarios

The background comparison algorithm is a widely used paradigm across various transportation fields, such as marine, urban, and railway [15,24,25]. It can be used to process video data and 3D point cloud data to separate the foreground and background. Its process is as follows: first, a clean background is constructed; then, the real-time collected data and the clean background are compared; next, filtering and clustering are performed based on a threshold, thereby identifying abnormal areas and data in the compared data. This method can effectively monitor the movement of abnormal objects.

Recently, the background comparison algorithm has been widely applied to 3D point cloud data collected in railway or highway environments. Filtering ground noise in urban road environments, comparing real-time 3D data with static background data, and clustering moving vehicles were proposed in [26]. Background data were further constructed based on range and intensity in [27], and the static template was subtracted from real-time point cloud data to identify and track vehicles. LiDARs are installed at railway crossings to collect 3D point cloud data and compare it with pre-prepared background data to identify passing trains in [28]. High-definition maps were used as background references in [29], and LiDAR data were compared with these maps to identify moving objects. Some studies combined background comparison algorithms with deep learning to improve the accuracy and robustness of object detection. A trained convolutional neural network model was employed in [30] to detect the projection of roadside point cloud data, and background comparison was further incorporated to enhance detection accuracy. A difference detection framework was proposed in [31] to process data collected from fixed LiDAR sensors, enabling precise and efficient tracking of moving persons. In railway scenarios, RGB images and point cloud data were integrated in [32], where background modelling across the two modalities was adopted to perform object detection.

**Figure 3:**
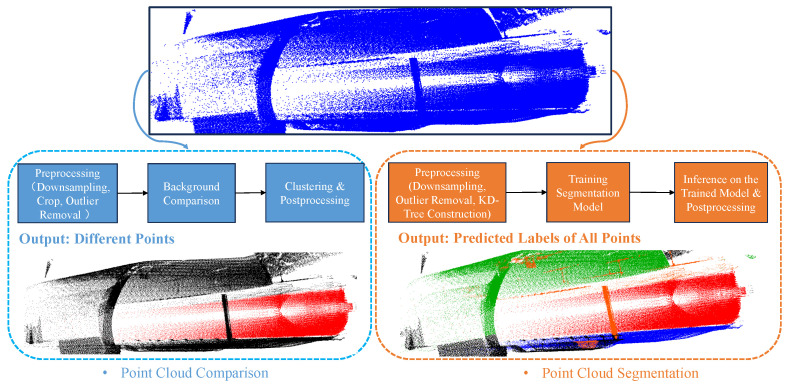
Point cloud comparison and segmentation methods for train detection.

In summary, the background comparison algorithm is a direct and efficient object detection framework that has demonstrated excellent performance across many transportation scenarios. However, it relies on a strong assumption: the background data is clean and does not change. Thus, the algorithm can clearly distinguish moving foreground from static background. However, in practical applications, noise from various sources often occurs. For example, in the train detection task based on point cloud data in this paper, the train point cloud often encounters noise from adverse weather conditions, which greatly affects the detection performance. This also prompts us to focus on deep learning-based point cloud segmentation models to achieve better generalization. The general flow and comparison of the two paradigms are shown in Figure 3.

### 2.3. Point Cloud Segmentation for Transportation Scenarios

Point cloud segmentation has always been a core task in intelligent transportation scenarios [33,34]. On one hand, it can provide dense three-dimensional semantic information, providing rich environmental understanding for various downstream tasks (such as object detection, obstacle recognition, scene reconstruction, etc.) [35,36]; on the other hand, as a visual perception task based on deep learning, point cloud segmentation usually has better robustness and generalization ability in adverse conditions such as rain, snow, haze, and night, thus having broad application prospects in practical engineering deployments [37,38]. For research on point cloud segmentation in traffic scenarios, existing work mainly focuses on two major directions: efficient segmentation in large-scale scenarios and improving the generalization ability of segmentation models across different scenarios and sensing conditions. In urban scenarios, a multi-scale network framework integrating point and voxel features was proposed in [39] to identify typical road traffic objects, including roads, vehicles, vegetation, and pedestrians. In railway scenarios, a multi-scale sparse three-dimensional convolutional network was designed in [40] to achieve high-precision railway track recognition, while point clouds were projected onto the image modality in [41], where a two-dimensional attention aggregation network was adopted for rail detection. Full-scene semantic segmentation for railway environments has also attracted increasing attention. A deep neural network framework incorporating point cloud downsampling, local and global feature extraction, spatial context aggregation, and regularization was proposed in [42] and validated on mobile laser scanning data. Point clouds collected from high-speed railways using unmanned-aerial-vehicle-borne LiDAR were further processed in [23], where massive point sets were compressed into compact graph structures preserving topological information through local spatial embedding. Gated integration graph convolutional networks were then employed for contextual segmentation, and an adaptive weighted cross-entropy loss was introduced to mitigate class imbalance. The above studies show that, even in railway scenarios with large-scale, complex three-dimensional environments, point cloud segmentation can still achieve good semantic parsing results.

In recent years, the release of large-scale railway point cloud datasets has also greatly promoted the development of point cloud semantic segmentation in railway scenarios. A multi-scenario railway point cloud dataset was constructed and made publicly available in [22], containing approximately 4.6 billion points annotated into 11 semantic categories. Similarly, a large-scale railway point cloud dataset was introduced in [43], where an active learning-based segmentation network was adopted to achieve weakly supervised semantic segmentation of railway point clouds.

Furthermore, point cloud semantic segmentation has also been combined with two-dimensional detection frameworks. A fusion architecture integrating a two-dimensional detector with a three-dimensional perception module was proposed in [44] to achieve real-time road vehicle detection and was successfully deployed in an intelligent traffic management platform. In contrast, in railway scenarios, most existing point cloud segmentation methods primarily focus on the semantic description of static targets (such as tracks, buildings, and infrastructure) in the scene and rarely integrate point cloud segmentation with moving obstacle or train detection. Based on the above observations, this paper further leverages detailed three-dimensional scene understanding from point cloud segmentation to design an efficient and robust train detection framework, enabling reliable identification and perception of train targets in railway scenarios.

## 3. Methodology

### 3.1. Problem Statement

The LiDAR sensors are usually installed on both sides of the railway to enable monitoring of the situation of a section of railway track, which is several tens of meters long. The input of the railway train detection task is point cloud data captured from these LiDAR sensors. Let P denote a railway-scene point cloud sample, where the input is represented as

(1) $$P={\left\{p_{i}\right\}}_{i=1}^{N}={\left\{\left(x_{i},y_{i},z_{i}\right)\right\}}_{i=1}^{N}\in R^{3\times N}.$$

where $\left(x_{i},y_{i},z_{i}\right)$ are the spatial coordinates of the points from railway scenes, and additional point-wise attributes, such as colour and intensity, can be incorporated when available. Since the industrial LiDAR sensors used in this work cannot capture colour information, only 3D spatial coordinates are used as input.

**Figure 4:**
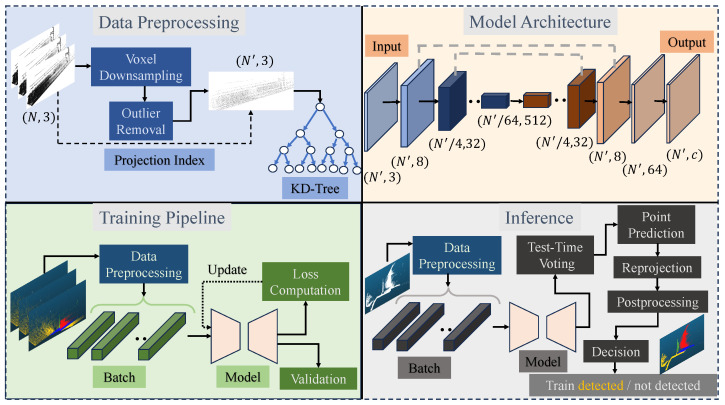
The overview of the proposed framework. The data pre-processing module takes the raw points, applies voxel downsampling and outlier removal, and produces a density-averaged set of sub-points. KD-tree structures are used for nearest-neighbour search. The model architecture is typically U-Net style, with encoder and decoder layers connected by skip connections. The entire training pipeline batches the processed point data, feeds it to the deep learning model, and then optimizes the model. In inference, test samples are fed into the frozen model to predict point labels. The final object-level decisions are generated by aggregating the point-level results.

As illustrated in Figure 4, the goal of the framework is to learn the parameters of the segmentation network in a supervised manner. Thus, for each railway-scene point cloud data, the semantic segmentation model can accurately predict the categories (rail, tunnel, wires, train, and other) at the point level. Then, after a post-processing step, the model would also decide whether there are trains at the frame level. Finally, the entire end-to-end framework must meet the requirements of actual railway safety surveillance scenarios: it should achieve a suitable inference speed, detect trains of different structures and sizes, and complete train detection under low illumination and bad weather conditions.

### 3.2. Pre-Processing

Given a raw LiDAR point cloud data, the first step is to process the data into a fixed railway corridor and regularize the point distribution before feeding it to the segmentation network. Specifically, we apply downsampling to obtain a more uniform density, remove isolated outliers caused by noise and moving objects, and construct a KD-tree for efficient neighbourhood queries used in both segmentation training and inference.

#### 3.2.1. Downsampling

Usually, the collected point cloud data will show a non-uniform distribution in the spatial domain. The point density is higher when near the LiDAR. To reduce redundancy and thereby enhance computational efficiency, we employed voxel downsampling to process the raw point cloud. Set a voxel size *s*, and the total space is separated into a regular voxel grid. Each point $p_{i}$ is assigned into a voxel index $v_{i}$, while, $V_{j}$ denotes the set of all points falling into the *j*-th voxel:

(2) $$v_{i}=\left\lfloor \frac{p_{i}}{s}\right\rfloor ,V_{j}=\left\{p_{i}|v_{i}=j\right\}.$$

All points in the same voxel would be represented by a computation-centric point $p_{j}$. And the downsampled point cloud data $P_{\mathrm{ds}}$ is shown as

(3) $$P_{\mathrm{ds}}=\left\{p_{j}|p_{j}=\frac{1}{|V_{j}|}\sum p_{i}\right\}.$$

where $|V_{j}|$ means the number of points in $V_{j}$.

#### 3.2.2. Outlier Removal

In the collected point cloud data, there are often very distant outliers, which can affect the search for the neighbourhood during model training and inference, disrupt the geometric consistency of the local structure, and thus degrade segmentation performance. We adopted a statistical outlier removal strategy based on the average distance of the neighbourhood to reduce the noise interference.

For each point $p_{j}\in P_{\mathrm{ds}}$, we find its *n* nearest neighbours $p_{k}\in N_{n}\left(p_{j}\right)$. The mean neighbourhood distance of $p_{j}$ is

(4) $$d_{j}=\frac{1}{n}\sum {∥p_{j}-p_{k}∥}_{2}.$$

If we set the distance threshold as $\sigma_{d}$, then a decision is made. Points with $d_{j}<\sigma_{d}$ are treated as inliers and reserved, whereas those with $d_{j}>\sigma_{d}$ are regarded as outliers and removed. This step can efficiently reduce noise interference from very distant outliers and improve the segmentation performance.

#### 3.2.3. KD-Tree Construction

After voxel downsampling and outlier removal, each frame of point cloud data still needs to construct a KD-tree to enable efficient neighbourhood queries via *k*-nearest neighbour (KNN) search, which is necessary for both model training and inference. During training, large-scale point cloud data is divided into multiple local regions. In each training epoch, multiple query points are selected, and a KNN search using the KD-tree is performed to obtain the local neighbourhoods of these points, which are then used as input to the neural network for training. This projection approach not only retains the local spatial relationships of the point cloud but also reduces memory constraints. Thus, it enables efficient training in multiple batches. During inference, after predicting each point, the predicted labels are re-projected back to the original positions of the full-resolution point cloud using the stored KD-tree index, thereby achieving point-level prediction.

### 3.3. Point Cloud Segmentation Network

Consistent with the standard U-net architecture, input points are processed by an encoder that reduces dimensionality through max pooling, followed by a decoder that restores dimensionality via upsampling. Skip connections link corresponding encoder and decoder layers. The decoder features are computed as a combination of the upsampled features from the current encoder layer and the features from the previous decoder layer, as described by the following formula:

(5) $$h_{d}^{\left(l\right)}=U\;\left(h_{e}^{\left(l\right)}\right)⊕h_{d}^{\left(l-1\right)}.$$

where $h_{d}^{\left(l\right)}$ is the decoder feature, U(·) represents the upsampling operation that transforms features to higher resolutions, $h_{e}^{\left(l\right)}\in R^{C}$ is the encoded feature, and $h_{d}^{\left(l-1\right)}\in R^{C}$ is the feature from the previous decoder layer.

Additionally, an attention-based pooling mechanism is employed to weigh the contributions of all features prior to the fully connected layers, thereby informing the final decision. This approach increases the influence of salient features while reducing the impact of less relevant features. The corresponding equation is

(6) $$\alpha =\delta \;\left(W_{a}h_{d}+b_{a}\right).$$

where α is the features to be pooled, δ is the Softmax function, $W_{a}$ and $b_{a}$ are learnable parameters.

The model is trained using a cross-entropy loss function defined as

(7) $$L=-\sum_{t=1}^{N_{t}}w_{t}\;y_{t}\;\log\left({\hat{y}}_{t}\right).$$

where $N_{t}$ denotes the number of classes, $w_{t}$ is the class weight, and $y_{t}$ and ${\hat{y}}_{t}$ represent the ground truth and predicted probabilities for class *t*. Inverse-frequency weighting was initially explored to address class imbalance; however, this approach resulted in a slight decrease in performance. Consequently, equal weights ($w_{t}=1.0$) were assigned to all classes.

### 3.4. Point-Level and Object-Level Train Detection

Our framework decomposes train detection into two stages: (i) point cloud semantic segmentation of the railway-scene point cloud data and (ii) object-level train aggregation, filtering, and decision-making. Formally, the proposed point cloud segmentation network $f_{\theta }\left(\cdot \right)$ is applied to predict point-level semantic probabilities, and a class-wise binarization is performed:

(8) $$S=f_{\theta }\left(P\right)\in {\left[0,1\right]}^{N\times C}.$$

where *C* is the number of data labels (0: rail; 1: tunnel; 2: wires; 3: train; 4: other), and $S_{t}\left(i,c\right)$ denotes the probability that point $p^{i}$ belongs to class *c*. The segmentation network is implemented in a deep learning architecture that aggregates local and global context features and is trained on annotated railway-scene point cloud data.

Based on the point cloud segmentation results, we performed post-processing steps to meet the requirements of our train detection task. The specific steps are shown in Algorithm 1. In our implementation, the train-point confidence threshold is set to $\tau_{\mathrm{train}}=0.6$. A clustering algorithm based on the Euclidean distance, with radius r=0.8 m, is used to aggregate high-probability predicted train points, and clusters containing fewer than M=1000 points are removed to suppress sparse noise responses. Thus, point-level predictions are translated to object-level instances. Then we calculate the minimum distance between each train instance and the rail. Instances whose distance exceeds the rail-distance threshold $\tau_{\mathrm{rail}}=2.0$ m are filtered out because they do not reflect the actual physical meaning. Finally, object-level verification is performed by jointly considering the number of points within each cluster and the geometric properties of its fitted 3D bounding box. Specifically, bounding box constraints Γ enforce minimum size requirements, including length ≥5 m, width ≥2 m, and height ≥2 m, to eliminate small spurious detections. Only the train instance that meets the point number and size requirements can be confirmed. Formally, an object-level train detection module $g_{\phi }\left(\cdot \right)$ clusters points assigned to train-related classes and produces instance-level detections:

(9) $$O=g_{\phi }\left(P,S\right)={\left\{\left({\tilde{N}}^{k},B^{k}\right)\right\}}_{k=1}^{K}.$$

Here, *K* is the number of detected trains in a frame, ${\tilde{N}}^{k}$ denotes the number of the *k*-th train, and $B_{t}^{k}$ denotes the corresponding 3D bounding box or instance mask of the *k*-th train. In our implementation, $g_{\phi }\left(\cdot \right)$ is a post-processing module that aggregates predicted train points and removes train instances with small size, low confidence, or locations inconsistent with the railway rail. The final detection decision is based on the point count and geometric size of each train instance.
**Algorithm 1** Post-processing for train detection**Require:** 
Railway point cloud P; segmentation network $f_{\theta }\left(\cdot \right)$; train class index $c_{\mathrm{train}}$; probability threshold $\tau_{\mathrm{train}}$; clustering radius *r*; cluster size threshold *M*; rail geometry R; rail-distance threshold $\tau_{\mathrm{rail}}$; bounding box constraints Γ**Ensure:** 
Point-level predictions ${\left\{{\hat{y}}_{i}\right\}}_{i=1}^{N}$; object-level train detection results O  1:Feed the raw data into the segmentation network:$$S=f_{\theta }\left(P\right)$$  2:**for** i=1 to *N* **do**  3:     ${\hat{y}}_{i}=\arg\max_{c}S\left(i,c\right)$  4:**end for**  5:Filter low-confidence train points:$$P^{\mathrm{train}}=\left\{p_{i}|S\left(i,c_{\mathrm{train}}\right)\geq \tau_{\mathrm{train}}\right\}$$  6:Apply clustering algorithms on $P^{\mathrm{train}}$ to obtain multiple train clusters ${\left\{C_{k}\right\}}_{k=1}^{K}$  7:Initialize detection set O  8:**for** each cluster $C_{k}$ **do**  9:    Compute number of points ${\tilde{N}}^{k}=\left|C_{k}\right|$10:    **if** ${\tilde{N}}^{k}<M$ **then**11:         continue12:    **end if**13:    Compute distance between rails and trains:$$d_{\mathrm{rail}}=\min_{p\in C_{k},\;r\in R}{∥p-r∥}_{2}$$14:    **if** $d_{\mathrm{rail}}>\tau_{\mathrm{rail}}$ **then**15:         continue16:    **end if**17:    Fit 3D bounding box $B^{k}=\mathrm{BBox}\left(C_{k}\right)$18:    **if** $B^{k}\notin \Gamma$ **then**19:         continue20:    **end if**21:    $O\leftarrow O\cup \{\left({\tilde{N}}^{k},B^{k}\right)\}$22:**end for**23:**return** $\left\{{\hat{y}}_{i}\right\}$, O

## 4. Experiments

**Figure 5:**
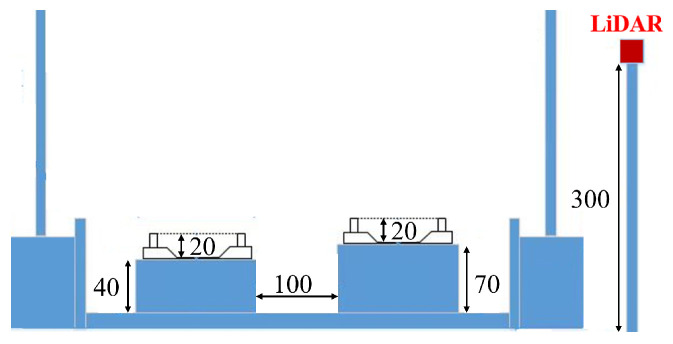
Railway-scene data collection schematic. All distances are in centimetres.

**Figure 6:**
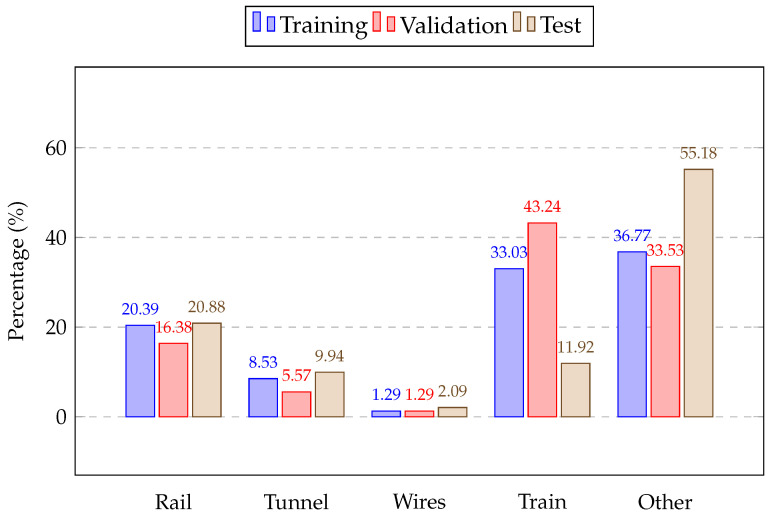
Point label distribution in training, validation, and test sets.

In this section, the evaluated datasets are first introduced, including how the data were collected in Figure 5 and the number of railway scenes, as well as the training, evaluation, and test data, and the class distribution of the labelled points is shown in Figure 6. Then, the implementation details of the proposed framework and the evaluation metrics are provided. The figure of the model training and validation losses is also shown to demonstrate the learning and generalization capabilities. In terms of quantitative results, we first compared the point cloud segmentation accuracy and efficiency of our proposed framework with those of other deep learning models. Then, we compared the train detection performance of our work with the industrial background comparison method. The ablation study of module parameters, such as the voxel size in the pre-processing step, batch size, KNNs in the deep learning model, and the probability threshold in the post-processing step, is also provided to demonstrate their influence on train detection performance. Finally, visualizations of detection results for various train configurations are presented, and robust, real-time train detection assisted by point cloud segmentation is discussed.

### 4.1. Datasets

LiDAR sensor: All data were collected using a Livox Mid-70 LiDAR sensor (Livox Technology Company Limited, Shenzhen, China). with a circular field of view of 70.4°, an effective detection range of up to 260 m, and a point rate of 100,000 points/s in single-return mode. The sensors were usually fixed at tunnel entrances and exits and mountain-side track segments.

Data size: The datasets comprise 19 scenes, with 258 training, 18 validation, and 1451 test samples. The test set includes 1095 clean background samples and 356 samples containing trains. It is worth noting that the validation dataset is not used to optimize model parameters. Instead, 18 samples are selected from unseen scenes that differ from the training data to evaluate cross-scene segmentation generalization. All quantitative results are evaluated on the test dataset. The differences in point distributions across the training, validation, and test sets allow us to evaluate the robustness of the model to realistic imbalance. Regarding point cloud annotation, we designed a complete hand-crafted pipeline. Using CloudCompare (version 2.13.0), multiple primary annotators labelled the data separately, after which a senior expert standardized and consolidated the annotations to ensure consistent and reliable ground truth.

### 4.2. Implementation Details and Evaluation Metrics

For performance evaluation, we conducted a quantitative analysis of the model across three aspects: overall segmentation performance, segmentation performance by classes, and train detection performance. The overall performance evaluation metrics include overall accuracy (OA), mean intersection over union (mIoU), and mean class accuracy (mACC), which evaluate the global semantic segmentation performance. For each class in the point cloud data, we calculated the IoU and accuracy to analyse the segmentation performance across different structural feature classes. Finally, since the train category is the most concerning aspect of our task, we also introduced precision, recall, and the F1-score as evaluation metrics. Precision reflects the false detection rate of the model on train targets, recall measures the missed detection rate on train targets, and the F1-score combines both to evaluate overall detection performance. The missed detection rate (MDR) and false detection rate (FDR) are used to evaluate sample-level train detection performance. If the intersection between the detected train and the GT (the true annotation) exceeds 40%, the train is considered correctly detected. The adoption of a 40% threshold is a balanced consideration between accuracy and robustness. In real railway scenes, due to the occlusion, limited view range, and the influence of sparse far-distance points, trains can only be detected partially. If the threshold is too high, the number of missed detections will increase. Meanwhile, if the threshold is too low, false alarms caused by noise or small wrong clusters would increase. Thus, based on the observation results, 40% is chosen as a moderate threshold. We further analysed all annotated train samples and observed that even the sparsest train point clouds contain more than 3000 points within the rail region. Thus, if there are no more than 1000 points of train predictions within the track area, it is regarded as no false alarm, which can reduce the risk of missed detections and suppress noise to a certain extent. All experiments were conducted using the PyTorch 1.13 framework on a single NVIDIA GeForce RTX 4090 GPU (NVIDIA Corporation, Santa Clara, CA, USA). Inference latency is measured at a single batch size during the testing stage. And the trainable parameters of the network are 4.994 million. The default parameter setting uses a voxel size of 0.2 m, 32 nearest neighbours for local feature aggregation, and a probability threshold of 0.1. Under this configuration, the test speed can reach 2.04 frames per second (FPS).

### 4.3. Training Loss and Validation Loss

**Figure 7:**
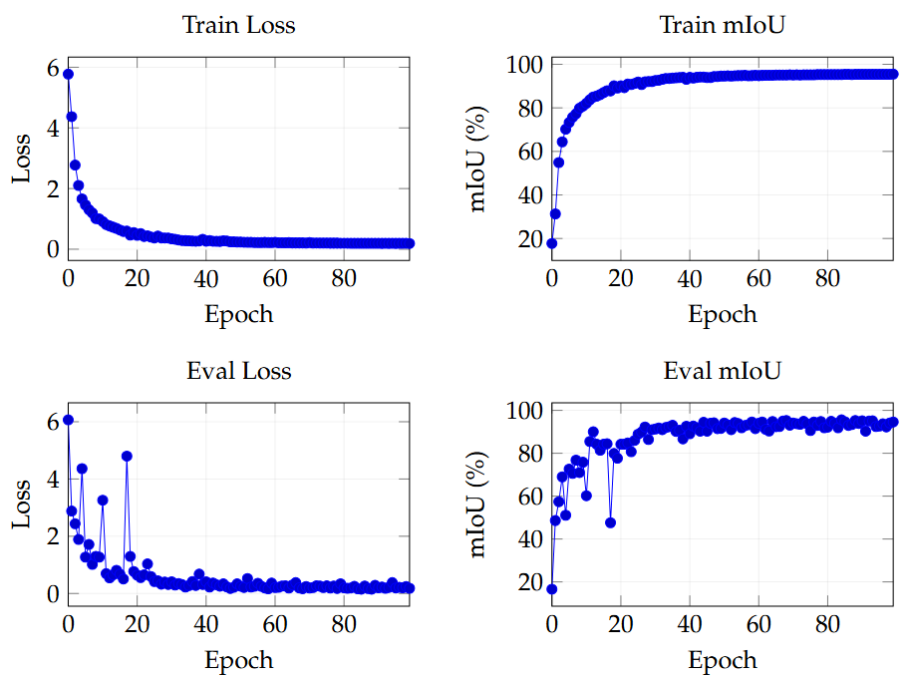
Training and validation curves across epochs.

The learning and generalization capabilities of this network can be seen from the training and evaluation losses and mIoU in Figure 7. Overall, the model has a strong learning ability and converges quickly. The evaluation curve shows good generalization. The train loss dropped rapidly from 5.8 at the beginning to 0.2. Train mIoU also steadily increased from 18.0% to 96.0%. This shows that the optimization process, including the design of the learning rate and loss function, is in the correct direction. The model exhibits high learning efficiency across tasks. In terms of generalization, both evaluation mIoU and train mIoU increase, with only a small gap between them, both around 95.0%. This shows that there is no obvious overfitting during training. The training and validation data show a consistent distribution. There were anomalous fluctuations in the validation metrics across several epochs. This is likely due to the small size of the validation set. We will consider expanding the validation set and conducting multiple evaluations to mitigate evaluation noise.

### 4.4. Segmentation Comparison with Other Point Cloud Segmentation Methods

Table 1 compares the quantitative segmentation performance of our proposed method with several representative point cloud segmentation methods, including KPConv [45], PointNet [46], and Point Transformer [47], on the railway-scene dataset. FPS is measured for segmentation inference only, without post-processing. Overall, the method we proposed achieved the best results on all global evaluation indicators. Specifically, the overall accuracy of the method proposed in this paper reaches 91.32%, the average intersection and union ratio reaches 75.94%, and the average category accuracy reaches 86.86%, all of which are significantly better than those of the compared point cloud segmentation methods. Especially, the significant improvement in mIoU and OA indicates that the network we designed can not only segment point cloud data with a large number of points but also achieve good segmentation results for point cloud data with complex structures.

For each category in the railway-scene data, we used IoU and accuracy to evaluate the structural and point segmentation results for each category. Considering that our task is train inspection, we have further added metrics such as precision, recall, and the F1-score to the train categories for evaluation. For the three categories of tracks, tunnels, and wires, the iou and acc of the method we proposed are significantly higher than those of the comparison methods, indicating that our method has higher accuracy and stability in fine-grained segmentation of railway point cloud scene data. In the wires category with sparse points, our method also achieved an accuracy of 81.78%. For the train segmentation results that we are most concerned about, the method we proposed achieved an IoU of 87.14%, an accuracy of 90.88%, a recall rate of 95.49%, and an F1-score of 93.13% in the train category. The higher the accuracy rate, the lower the risk of false alarms by trains; the higher the recall rate, the lower the risk of missed alarms by trains.

In terms of detection efficiency, our model’s reasoning speed reaches 2.35 FPS, which is significantly higher than that of the Point Transformer [47] and slightly higher than that of KPConv [45]. Although it is lower than the PointNet [46] method, our method can achieve an inference speed close to real time while maintaining segmentation accuracy, forming a relatively ideal balance between performance and efficiency, making it suitable for the deployment of actual railway train detection applications.

### 4.5. Train Detection Comparison with the Industrial Background Comparison Method

Table 2 presents the quantitative results of the comparison-based and segmented methods on the train detection task. FPS is measured for the complete detection pipeline, including post-processing. The entire test set contains 1451 cloud data from railway yard sites, of which 356 contain trains, and 1095 contain clean background samples. The segmented method we proposed outperforms the comparison-based method across both the missed detection rate and false detection rate, the two indicators of detection accuracy. Specifically, the MDR based on the segmentation method is 0.76 %, which is lower than 3.31 % based on the comparison method. The FDR based on the segmentation method is 1.31 %, which is also lower than 2.76 % based on the comparison method. These results demonstrate that the segmented method achieves higher accuracy and robustness in train detection tasks in actual railway environments than the contrastive method. Although the segmentation method achieves a reasoning speed of 1.44 FPS, which is lower than the 3.24 FPS of the contrast method with a simple algorithm, it offers clear advantages in detection accuracy and can still meet the requirements of practical applications.

In addition to quantitative results, the segmentation-based method also offers other advantages in practical applications. Firstly, this method is more robust. By directly performing semantic modelling on point clouds, the model can effectively avoid interference from rain and fog noise and LiDAR displacement. Secondly, this method is highly automated and can support a fully automated process from model deployment to point cloud processing and train detection, thereby enhancing the method’s engineering usability. Finally, the segmentation-based detection framework can learn features of semantic categories in railway scenarios. Not only can it effectively distinguish the train from the background, it can also stably detect trains of different types, shapes, and sizes, thereby demonstrating stronger generalization ability.

### 4.6. Analysis of Train Detection in Rainy Weather Conditions

Adverse weather conditions, such as rain and fog, can increase noise interference in LiDAR-collected point cloud data. Noise is caused by reflections from raindrops in the air and interference from near-surface rain. To evaluate the robustness of the proposed segmentation-based train detection method under rainy conditions, this section presents a systematic analysis of the detection results from both qualitative and quantitative perspectives.

**Figure 8:**
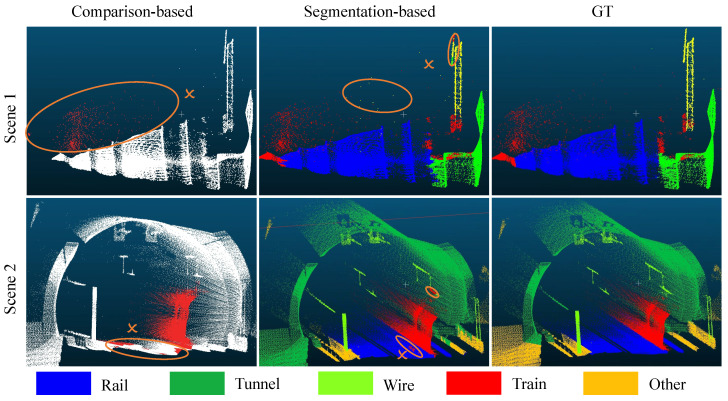
Visualization of comparison-based and segmentation-based train detection under rain conditions. Circle and cross symbols donate false detection. In scene 1, the comparison-based method would treat rain noise as a train and thus classify it as a false detection. In scene 2, the comparison-based method also treats near-surface rain as trains.

Figure 8 presents the detection results obtained by training on rainy days with comparison-based and segmentation-based methods. It is worth noting that, due to limited semantic understanding of point cloud data, the contrastive method for train detection under adverse weather conditions may misclassify air and near-ground noise as trains, resulting in a large number of false detections. This indicates that the contrastive method imposes extremely stringent requirements on environmental background consistency, thereby increasing the difficulty of real-world engineering deployment. In contrast, the segmentation-based method can detect semantic and geometric features in point cloud data and is highly resistant to rain and fog noise, thereby reducing false alarms. To further evaluate the robustness of the segmentation methods under adverse weather conditions, 227 false alarm samples from the industrial background comparison method are collected for a supplementary experiment. All of these samples detected trains, but manual inspection revealed none. We fed these samples into the segmentation-based framework for inference. The results show that no trains were detected, indicating that the proposed method can effectively suppress false alarms caused by rain noise, which provides additional quantitative evidence for the robustness of the proposed framework.

Table 3 presents the quantitative results of the segmentation method for sunny and rainy days. Under rainy conditions, although segmentation performance decreased slightly due to noise interference, it remained relatively stable overall and during train inspection tasks. OA and mACC are 94.00% and 92.77%, respectively, and IoU in the train set is 94.01%. The precision rate is 99.09%, the recall rate is 94.83%, and the F1-score is 96.91%. This indicates that the overall quality of point cloud segmentation remains robust under adverse weather conditions. This also indicates that the segmented method can reduce interference from rain and fog noise and is more suitable for train detection in complex railway environments.

### 4.7. Ablation Study

#### 4.7.1. Analysis of Voxel Size and KNNs

As shown in Table 4, to analyse the influence of key parameter settings on the performance and efficiency of point cloud segmentation, we evaluated segmentation results and inference speeds across different voxel sizes and KNN neighbourhood sizes. Compared with voxel sizes of 0.6, the segmentation performance of the model is significantly improved not only in global metrics (OA, mACC, and mIoU) but also in class-level segmentation accuracy, when voxel sizes are 0.06 or 0.2. Therefore, although the model has a relatively fast inference speed at a voxel size of 0.06, the excessive loss of geometric details and local structural information prevents it from fully mining the semantic information of the point cloud data. When the voxel size is 0.2, the model achieves 2.04 FPS, faster than when the voxel size is 0.6, and it does not lag behind in global metrics. Moreover, in train detection, even its performance improved. It is indicated that when the voxel size is 0.06, there is substantial redundancy in the point cloud data. From the perspective of the KNN neighbourhood size, when KNN is set to 8, the segmentation performance is the lowest. This is because the local feature information is insufficient, preventing the model from fully capturing the structural context features. When KNN is set to 16, the segmentation performance of the model significantly improves. When the number of KNN neighbourhoods is increased to 32, the overall segmentation performance and the evaluation metrics for the train category are highest. Among them, mIoU reached 70.96%, and the F1-score of the train category increased to 94.98%.

Based on the above analysis, both voxel size and the number of KNN neighbourhoods would influence the performance and computational efficiency of point cloud segmentation. Smaller voxel sizes and larger neighbourhood sizes can improve segmentation accuracy, but they will also increase computational costs. On the contrary, although a larger voxel size and a smaller number of neighbourhoods increase the inference speed, they sacrifice the segmentation accuracy. Based on the experimental results, the subsequent experiments in this paper adopt a voxel size of 0.2 and a KNN neighbourhood size of 32 to achieve the best trade-off between segmentation accuracy and operational efficiency.

#### 4.7.2. Analysis of Inference Time and Probability Threshold

In the inference, the model uses a minimum-sampling-threshold parameter based on the voting mechanism to predict the point cloud. This parameter can be used to measure the degree of point cloud sampling during testing. The higher the threshold, the more thorough the sampling. Table 5 presents the comparison results of point cloud segmentation and train detection performance under different probability threshold settings. When the probability threshold is set to 0.01, despite having the fastest inference speed, the overall segmentation performance is the lowest. This indicates that the threshold is set too low, leading to early termination of the sampling process and, in some scenarios, wrong point cloud prediction, thereby reducing the segmentation quality of the model. As the threshold increases to 0.1 and 1, the model shows similar overall segmentation performance and detection metrics across the train categories. This indicates that a moderate probability threshold can ensure the stability of the prediction. However, when the threshold is 1, the inference speed drops significantly to only 1.11 FPS. This is because a higher threshold requires greater sampling coverage, thereby increasing the computational cost of the model. Overall, a probability threshold that is too low will lead to unstable predictions, while one that is too high will affect system efficiency. Based on the trade-off between segmentation accuracy and computational efficiency, we set the probability threshold to 0.1 in the experiment to achieve the best performance.

### 4.8. Visualization of Train Detection and Discussion

**Figure 9:**
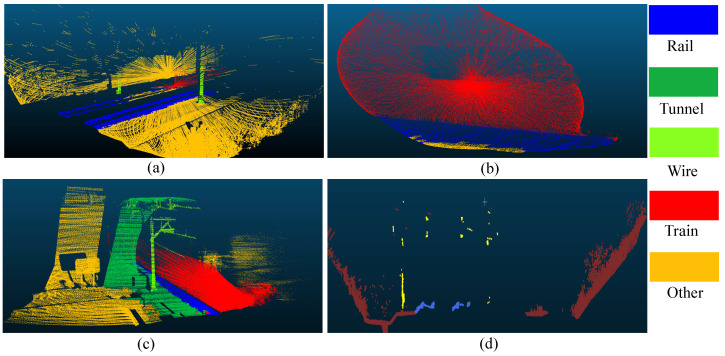
Visualization of the detected trains. (**a**) Sparse train point cloud sample. (**b**) Dense train point cloud sample. (**c**) High-speed train point cloud sample. (**d**) Clean background point cloud sample.

Figure 9 shows the proposed train detection visualization results in different railway scenarios. As shown in Figure 9a,b, our method achieves stable detection results under both sparse and dense train point cloud conditions. As shown in Figure 9c, high-speed trains can also be accurately identified, indicating that our proposed method can achieve efficient detection performance across different point cloud densities, structures, and sizes. Furthermore, Figure 9d shows the detection results for the clean background point cloud data sample. The model did not get false train predictions and proves its stability in real-world applications.

It is worth mentioning that the proposed method in this paper has been deployed and used in the actual railway detection system. It can consistently and accurately output train detection results during long-term, continuous, real-world operation, demonstrating the engineering practicability of this method. However, the method still has some limitations. In real-world applications, we have found that point cloud data collected across different scenarios have significant coordinate system differences, leading to inconsistent spatial distributions of point clouds and causing feature offset problems, which in turn have a certain impact on the results of point cloud segmentation and train detection. Future work will focus on standardizing LiDAR data acquisition and coordinate system definitions to improve system performance.

## 5. Conclusions

In this paper, a railway train detection framework based on segmentation is proposed. This framework utilizes large-scale LiDAR point cloud data to detect trains. By translating the traditional background comparison detection method into a semantic segmentation model with geometry-aware post-processing, this proposed method enhances robustness against background interference, rain noise, and changes in point density. Extensive experiments have demonstrated that this framework achieves high segmentation accuracy and reliable object-level target detection across various scenarios and under difficult conditions. An ablation study further confirmed that, under the default configuration, this framework maintains actual computational efficiency, thereby proving its feasibility for real-time railway monitoring applications.

The proposed framework requires installing and fixing LiDAR sensors on one side of the railway and acquiring geometric information of the railway to constrain post-processing. Its performance also depends on sufficient point density and reasonable parameters. Sparse point clouds, occlusions, and highly unstructured environments may affect the detection results. This framework is used for a railway safety surveillance system that requires efficient, stable train detection. It can be used for large railway scenario monitoring, real-time train management, and obstacle detection at key points. Further work would evaluate more point cloud data from railway scenes to enhance the robustness of the proposed method and further improve the efficiency of the segmentation network, making it suitable for large-scale deployments.

## Figures and Tables

**Table 1:** Quantitative segmentation results of different approaches. Best results are shown in bold.

Methods	OA	mIoU	mACC	Rail		Tunnel		Wires	
				IoU	ACC	IoU	ACC	IoU	ACC
KPConv	81.17	66.54	82.02	66.76	85.10	42.78	75.18	64.06	73.71
PointNet	80.58	54.05	72.13	56.31	76.44	32.09	71.81	33.71	46.68
Point Transformer	85.01	64.30	80.43	67.15	83.00	36.54	71.51	61.72	73.22
Ours	**91.32**	**75.94**	**86.86**	**75.24**	**87.38**	**64.74**	**77.68**	**64.89**	**81.78**

Methods	Train					Other		FPS	
	IoU	ACC	Precision	Recall	F1	IoU	ACC		
KPConv	77.36	90.48	84.21	90.48	87.24	81.74	85.64	2.07	
PointNet	72.22	83.38	84.37	83.38	83.87	75.92	82.36	**2.81**	
Point Transformer	74.91	89.17	82.41	89.17	85.66	81.17	85.25	0.96	
Ours	**87.14**	**95.49**	**90.88**	**95.49**	**93.13**	**87.69**	**91.98**	2.35	

**Table 2:** Train detection results comparison. All methods are evaluated on the same test dataset containing 356 train samples and 1095 background samples.

Method	MDR	FDR	FPS
Comparison-based	3.31%	2.76%	3.24
Segmentation-based	0.76%	1.31%	1.44

**Table 3:** Railway-scene point cloud data segmentation results under different weather conditions. The sunny and rainy subsets each contain 125 samples from 7 unseen scenarios, different from the training data, for cross-scene evaluation.

Weather	OA	mIoU	mACC	Train				
				IoU	ACC	Precision	Recall	F1
Sunny	96.82	92.36	95.46	98.87	99.89	98.98	99.89	99.43
Rainy	94.00	82.00	92.77	94.01	94.83	99.09	94.83	96.91

**Table 4:** Segmentation results with different voxel sizes and KNNs.

Parameters	OA	mIoU	mACC	Train					FPS
				IoU	ACC	Precision	Recall	F1	
Voxel Size = 0.06	86.85	69.96	84.92	87.07	87.74	99.13	87.74	93.09	0.41
Voxel Size = 0.2	86.77	70.96	86.46	90.43	90.94	99.38	90.94	94.98	2.04
Voxel Size = 0.6	80.52	60.58	80.27	79.92	80.94	98.44	80.94	88.84	5.51
KNN = 8	79.22	60.93	78.03	76.89	79.18	96.38	79.18	86.94	3.37
KNN = 16	84.15	67.41	83.75	83.27	83.98	99.01	83.98	90.87	2.84
KNN = 32	86.77	70.96	86.46	90.43	90.94	99.38	90.94	94.98	2.04

**Table 5:** Segmentation results with different probability thresholds.

Parameters	OA	mIoU	mACC	Train					FPS
				IoU	ACC	Precision	Recall	F1	
Probability = 0.01	85.49	69.43	86.02	87.77	88.50	99.06	88.50	93.48	2.26
Probability = 0.1	86.77	70.96	86.46	90.43	90.94	99.38	90.94	94.98	2.04
Probability = 1	86.67	70.74	86.44	90.55	90.98	99.48	90.98	95.04	1.11

## Data Availability

The data presented in this study are available on request from the corresponding author. The data are not publicly available due to confidentiality agreements with the data provider.
